# Insights into the molecular-level effects of atmospheric and room-temperature plasma on mononucleotides and single-stranded homo- and hetero-oligonucleotides

**DOI:** 10.1038/s41598-020-71152-1

**Published:** 2020-08-31

**Authors:** Liyan Wang, Hongxin Zhao, Dong He, Yinan Wu, Lihua Jin, Guo Li, Nan Su, Heping Li, Xin-Hui Xing

**Affiliations:** 1 MOE Key Laboratory for Industrial Biocatalysis, Department of Chemical Engineering, Center for Synthetic and Systems Biology, Tsinghua University, Haidian District, Beijing, 100084 People’s Republic of China; 2 Biobreeding Center, Wuxi Research Institute of Applied Technologies, Tsinghua University, Wuxi, 214072 People’s Republic of China; 3 TmaxTree Biotechnology Co. Ltd., Luoyang, 471023 People’s Republic of China; 4 Zhejiang Province Key Laboratory of Plant Secondary Metabolism and Regulation, College of Life Sciences and Medicine, Zhejiang Sci-Tech University, Hangzhou, 310018 People’s Republic of China; 5 College of Bioengineering, Beijing Polytechnic, Beijing, 100176 People’s Republic of China; 6 Department of Engineering Physics, Tsinghua University, Haidian District, Beijing, 100084 People’s Republic of China; 7 Center for Synthetic and System Biology, Tsinghua University, Beijing, People’s Republic of China; 8 Institute of Biopharmaceutical and Health Engineering, Tsinghua Shenzhen International Graduate School, Shenzhen, 518055 People’s Republic of China

**Keywords:** Biological techniques, Mutation

## Abstract

Atmospheric and room-temperature plasma (ARTP) has been successfully developed as a useful mutation tool for mutation breeding of various microbes and plants as well animals by genetic alterations. However, understanding of the molecular mechanisms underlying the biological responses to ARTP irradiation is still limited. Therefore, to gain a molecular understanding of how irradiation with ARTP damages DNA, we irradiated the artificially synthesized mononucleotides of dATP, dTTP, dGTP, and dCTP, and the oligonucleotides of dA_8_, dT_8_, dG_8_, dC_8_, and dA_2_dT_2_dG_2_dC_2_ as chemical building blocks of DNA with ARTP for 1–4 min, identified the mononucleotide products using ^31^P- and ^1^H-nuclear magnetic resonance spectroscopy (NMR), and identified the oligonucleotide products using matrix-assisted laser desorption/ionization time-of-flight mass spectrometry (MALDI-TOF MS) during ARTP treatment. The observed ^31^P-and ^1^H**-**NMR spectrum signals for the plasma-treated and untreated mononucleotides indicated that dATP was less stable to plasma irradiation than the other mononucleotides. The oligonucleotides after treatment with ARTP were found to have been broken into small fragments as shown by mass spectrometry, with the cleaved bonds and produced fragments identified according to their expected spectral *m/z* values or molecular weights derived from their *m/z* values. The stabilities of the oligonucleotides differed to ARTP irradiation, with dT_8_ being the most stable and was more beneficial to stabilizing single-stranded oligonucleotide structures compared to the other base groups (A, G, and C). This was consistent with the average potential energy level obtained by the molecular dynamic simulation of the oligonucleotides, i.e., dT_8_ > dC_8_ > dA_8_ > dG_8_ > dA_2_dT_2_dG_2_dC_2_. In summary, we found that ARTP treatment caused various structural changes to the oligonucleotides that may account for the wide and successful applications reported for ARTP-induced mutation breeding of various organisms.

## Introduction

In the past decade, the effects of so called cold atmospheric plasma (CAP) such as atmospheric pressure glow discharge (APGD) plasma on biological phenomena have attracted great interest^1–3^. Extensive research has shown that APGD plasma causes DNA damage in a dose-dependent manner in both prokaryotic and eukaryotic cells^1–4^. Hence, applications, including cancer and dental treatments, surface sterilization, surface modification of biocompatible materials, and surgery, have been developed^5–18^. APGD plasma can be generated at moderate temperatures (≤ 50 ºC)^6^, and therefore it has been exploited to induce mutagenesis for mutational breeding^1,4^.

**Figure 1:**
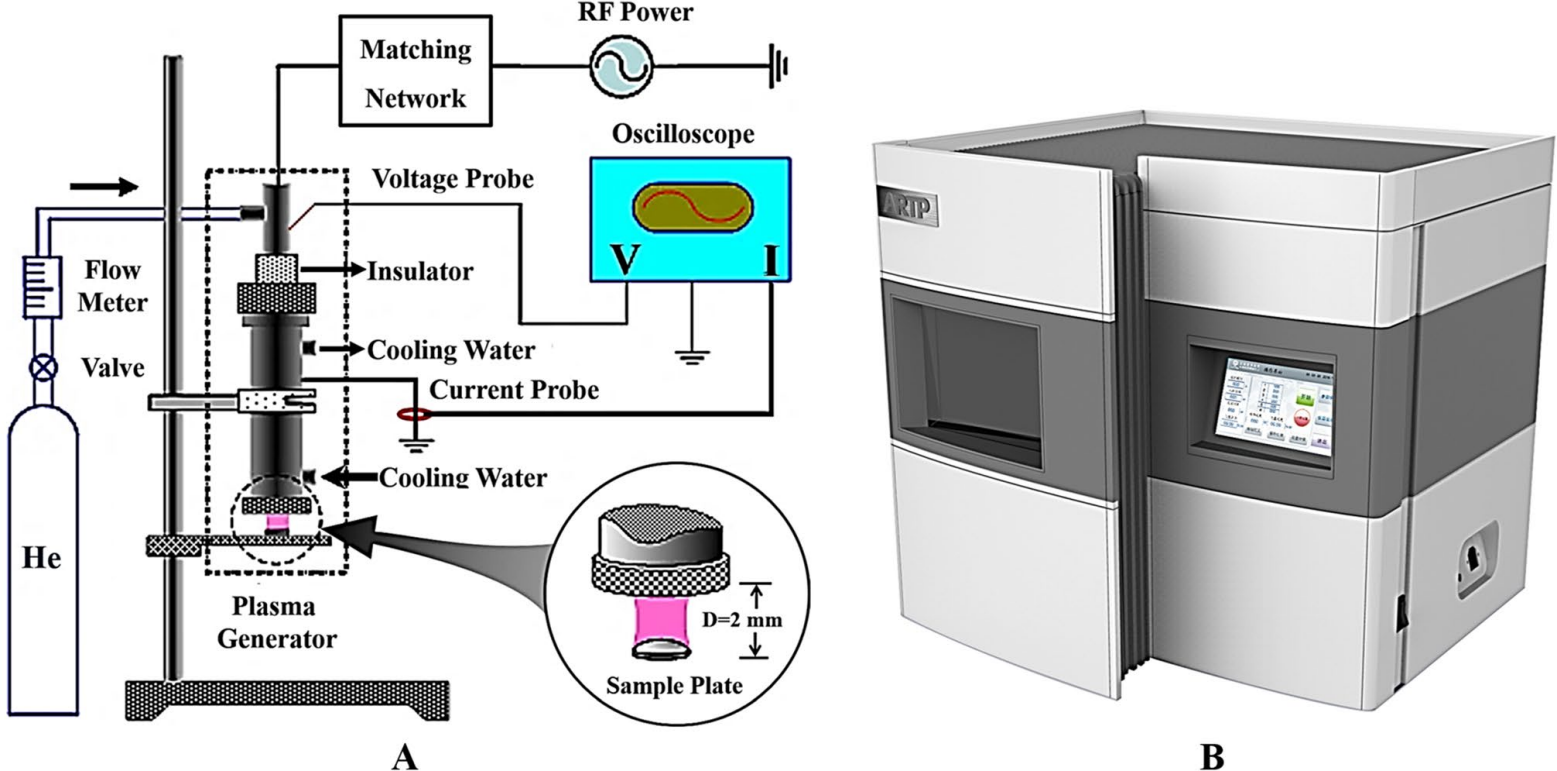
(**A**) Schematic of the ARTP-generating and -dosing instrumentation [(D) stand-off distance; RF, radio-frequency]; (**B**) Outlook of the instrument.

Previously, based on APGD, we developed an “atmospheric and room-temperature plasma (ARTP)” driven by a radio-frequency power supply and equipped with water-cooled bare-metallic copper electrodes (Fig. 1). We have employed this system to mutate the genomes of various microbes and plants as well as animals, including bacteria, microalgae, fungi, and yeast^1,4, 19–24^. The results of those studies indicated that ARTP mutation is a rapid, effective, convenient, and multifaceted means of generating mutant libraries with sufficient diversity for the improvement of phenotypes. The mechanism(s) behind ARTP mutagenesis is (are) presumably related to the actions of reactive oxygen species (ROS)/reactive nitrogen species (RNS) generated by the reaction of plasma with the water environment of ARTP**-**treated cellular samples. These reactive species damage the cellular DNA and, consequently, induce the activation of repair systems such as the bacterial SOS repair system^4,25^. Since it was first developed, our ARTP mutation instrument has been successfully used to perform mutation breeding for more than 100 organisms^1^. By quantitatively comparing the extent of DNA damage and mutation rates induced by different types of mutagens, including ARTP, 4-nitroquinoline-1-oxide plus N-methyl-N′-nitro-N-nitrosoguanidine (MNNG), and ultraviolet radiation, ARTP was found to be a powerful mutagen with which to improve the organism characteristics^26^.However, the detailed molecular mechanism(s) by which ARTP damages DNA remains unclear.

To gain insights into the molecular mechanisms by which CAP damages DNA molecules, previous studies examined the reactivities of ROS and RNS toward nucleobases. Other studies focused on the effects of CAP or ARTP irradiation on isolated DNA molecules or analogs and then characterized them by gel electrophoresis, molecular combing, Fourier transform infrared spectroscopy, Raman spectroscopy, matrix-assisted laser desorption ionization-time of flight mass spectrometry (MALDI-TOF/MS), and/or high-performance liquid chromatography electrospray ionization-mass spectrometry (HPLC–ESI–MS/MS)^4,27,28^. In the aforementioned studies, structural comparison of plasma-treated and untreated DNA molecules or analogs demonstrated that irradiation with plasma can result in DNA fragmentation. The effects of helium-based ARTP on plasmid DNA have also been investigated in our previous work^26–28^. However, details of the structural changes and base-breakage patterns during the DNA-damage process(s) by plasma are still unknown.

For the work reported herein, to investigate the damage pattern of DNA by ARTP treatment at molecular level, we developed a method employing plasma-based breakage of DNA bases in mono-, oligo-, and polynucleotides using the artificially synthesized mononucleotides of dATP, dTTP, dGTP, and dCTP, and the five oligonucleotides of dA_8_, dT_8_, dG_8_, dC_8_, and dA_2_dT_2_dG_2_dC_2_, and treated them by a previously developed, helium-based ARTP system^4^. After treatment of the oligonucleotides with ARTP for different time periods, the products were characterized by ^31^P- and ^1^H-NMR spectroscopy, and MALDI-TOF/MS to identify chemical changes induced by irradiation with ARTP. The molecular dynamic simulation was also performed using Discovery Studio v2.5 (DS) to estimate the stability of the synthesized oligonucleotides.

### Results

#### ARTP-induced effects on the molecular structures of mononucleotides

A mononucleotide (dNTP) consists of three major parts: a nitrogen base, a sugar moiety, and a triphosphate. Only the nitrogen bases differ between the four mononucleotides. NMR is a powerful analytical tool to determine the structures of organic compounds by analyzing the environments of ^1^H, ^13^C, and ^31^P nuclei. Thus, acquisition of ^31^P-and ^1^H-NMR spectra of the plasma-treated mononucleotides was helpful in determining the chemical and structural changes.

**Figure 2:**
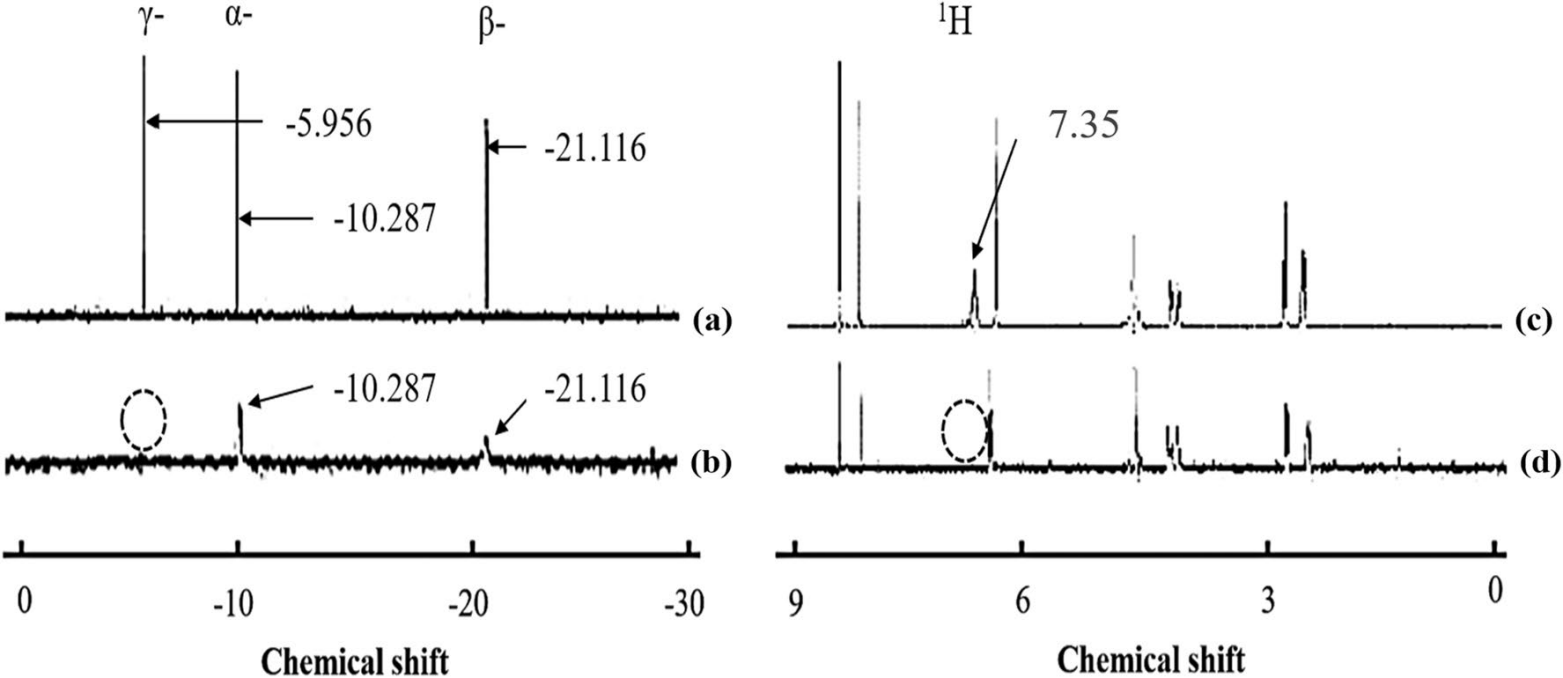
^31^P- and ^1^H-NMR spectra of dATP before and after ARTP-treatment 2 min (D_2_O as solvent to dissolve samples). (**a**,**b**) ^31^P-NMR spectra of dATP before (**a**) and after (**b**) ARTP treatment. (**c**,**d**) ^1^H-NMR spectra of dATP before (**c**) and after (**d**) ARTP treatment. The signals at δ = − 6.0, − 10.3 and − 21.1 are those of the γ-, α- and β-phosphates.

The ^31^P-NMR spectra of dATP before and after ARTP treatment are shown in Fig. 2a,b. The chemical shifts (δ) at − 6.0, − 10.3, and − 21.1 in the ^31^P-NMR spectrum are those of the γ-, α- and β-phosphates, respectively. The γ-phosphate group of dATP disappeared after 4-min treatment with ARTP, and the α- and β -phosphate groups also were cleaved when treated for an additional time (Fig. 2a,b). Structural changes related to the deoxyribose moieties and bases were also seen in the ^1^H-NMR spectra (Fig. 2c,d). The signal at δ = 7.35 in the spectrum of dATP [Fig. 2c; Fig. S1(2)], which is that for the hydrogens of the amino group at C6 of adenine, disappeared quickly and completely upon treatment with plasma (Fig. 2d). Actually as a chemical change, the δ = 7.35 signal in the spectrum as time went by also disappeared slowly because the NH_2_ group was going to exchange with ND_2_ in deuterium oxide. The signal at δ =  − 0.51 in the ^1^H-NMR spectrum of dCTP was a new peak after treated by ARTP [Fig. S1(4)]. It also indicated that the structure of dCTP has changed. Interestingly, the plasma-induced structural changes in dGTPs and dTTPs by ARTP treatment were not as obvious as dATP and dCTP from the ^1^H-NMR spectrum [Fig. S1(6),(8)], and the reason would be discussed latter.

The above results suggested that the phosphate groups as one of chemical groups of the dATP and dCTP mononucleotides were relatively susceptible to cleavage upon ARTP treatment than other chemical groups. Notably, deoxyribose and the individual, isolated bases were stable to ARTP treatment. However, the stability of the deoxyribose moiety after treatment with ARTP decreased when covalently bonded to a triphosphate via a phosphodiester bond (Fig. S1). These NMR spectra indicated that the chemical natures of the mononucleotides determined their stability to irradiation with ARTP (Fig. S1).

### Structural changes of the oligonucleotides induced by ARTP treatment

**Figure 3:**
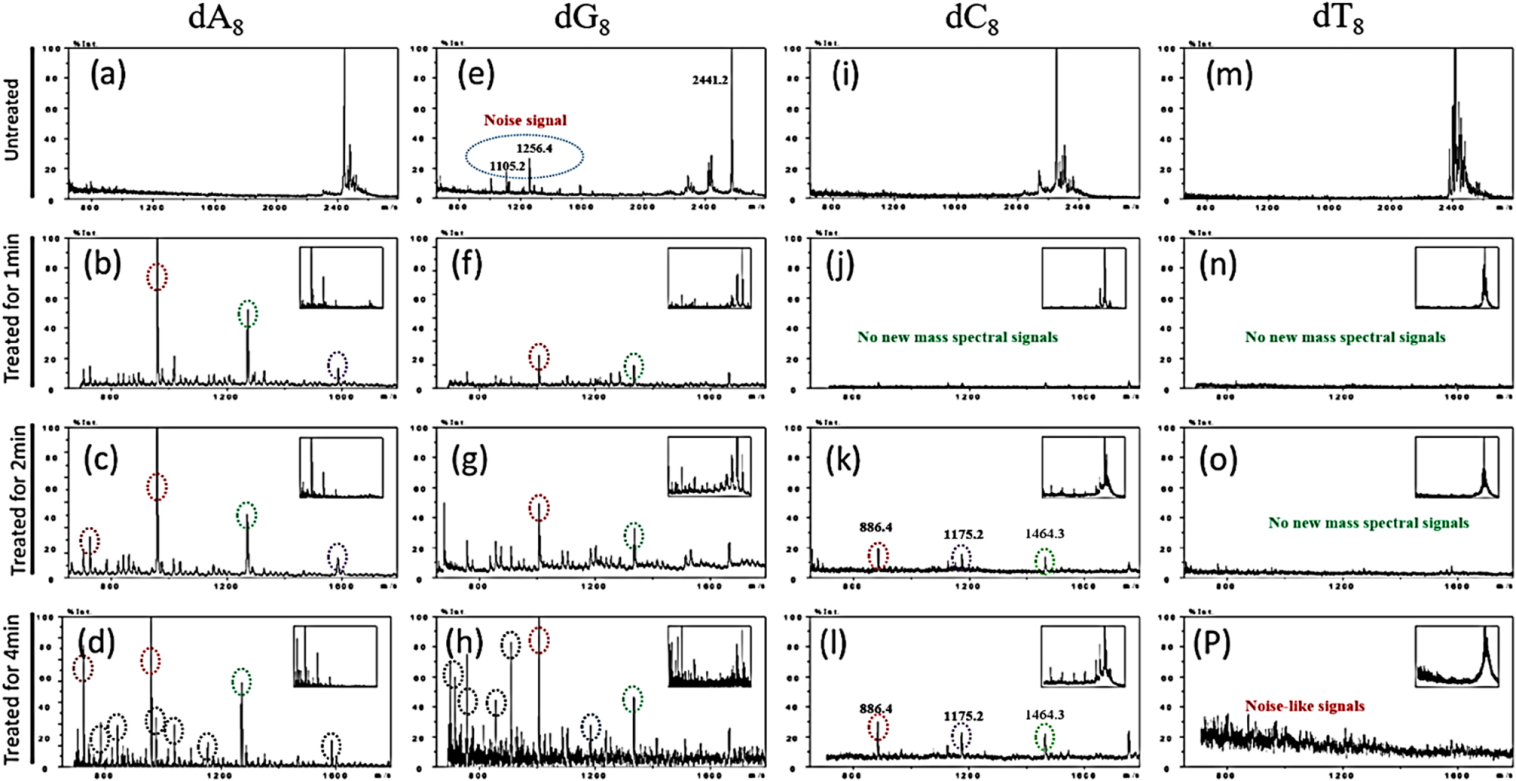
MALDI-TOF/MS of the oligonucleotides. The mass spectra were acquired over an *m*/*z* range of 680–2,800. To clearly display the spectral regions containing the nucleotide fragments present after ARTP treatment, the spectral region between *m*/*z* 680 and *m*/*z* 1,600 is shown in the larger diagrams with the complete spectra shown in the inserts. The image of mass spectrogram of 680 ~ 2,800 (m/z) is shown in the upper right corner.

The changes in the oligonucleotides induced by ARTP treatment were characterized by MALDI-TOF/MS. To correct for background noise, the mass spectrum of the solvent, distilled water, was acquired in parallel (Fig. S2). Mass peaks were found for the distilled water sample when the *m*/*z* ratio was < 650 (Fig. S2), whereas almost no signals were observed when the *m*/*z* ratio was > 680 (Fig. S2). Therefore, the mass spectra of dA_8_, dG_8_, dC_8_, and dT_8_ were acquired between *m*/*z* values of 680 and 2,800 (Fig. 3a–p). Except for two noise signals with *m*/*z* values of 1,256.4 and 1,105.2 in the spectrum of dG_8_ (Fig. 3e), no other noise signals were observed that might reflect the presence of contaminants. The lack of extraneous signals indicated that the oligonucleotides were of high quality and suitable for structural analysis by MALDI-TOF/MS (Fig. 3a,e,i,m).

After dA_8_ and dG_8_ had been treated with ARTP for 1, 2, or 4 min, several different signals were detected in their mass spectra (Fig. 3b–d,f–h) respectively. The oligonucleotides were degraded into smaller fragments upon treatment with plasma. Four new peaks appeared at different m/z positions (Fig. 3b–d). Four new signals at small *m/z* increased in intensity with increase of ARTP treatment time in the spectrum of dA_8_, whereas the signal of intact dA_8_ (*m*/*z* = 2,441.2) had disappeared from 2 min ARTP treatment (Fig. 3c,d). Similar results, which several new peaks appeared at different m/z position were obtained from the spectra of dG_8_ (Fig. 3f–h). And signals’ intensity in the spectrum increased with time treated by ARTP (Fig. 3f–h). Moreover, all signal intensities corresponding to products formed upon treatment of dA_8_ and dG_8_ with ARTP increased with time. This result suggested that more new fragments were formed along with the prolonged treatment time for poly A and G (Fig. 3b–d,f–h). These results clearly showed that poly A and G were broken rapidly with increase of ARTP treatment time, but the quantitative analysis for the oligonucleotide breakage pattern upon ARTP treatment needs to be performed further.

Interestingly, no new mass spectral signals were detected for dC_8_ after an ARTP treatment of 1 min. When dC_8_ was treated for 2 min and 4 min, three new signals with weak intensities were detected at *m*/*z* = 886.4, 1,175.2, and 1,464.3 (Fig. 3k,l). These observations suggested that dC_8_ was more resistant to ARTP-induced fragmentation than dA_8_ and dG_8_ under the same conditions. Concerning dT_8_, no MALDI-TOF/MS signals associated with new fragments were observed after dT_8_ had been treated with ARTP for 1–2 min (Fig. 3n,o). When treatment was increased to 4 min, however, the signal intensity of intact dT_8_ decreased somewhat, and several very weak, noise-like signals were observed. Therefore, dT_8_ was more stable to ARTP treatment, as hardly any fragments smaller than the original ion were consistently seen (Fig. 3p).

In summary, the several bonds within the oligonucleotide structures were broken by ARTP treatment, with the adenine (A) and guanine (G) purine bases being less stable to ARTP than the cytosine (C) and thymine (T) pyrimidine bases. In addition, dT_8_ was the most stable to ARTP treatment.

### Identification of the oligonucleotide fragments generated by ARTP treatment

**Figure 4:**
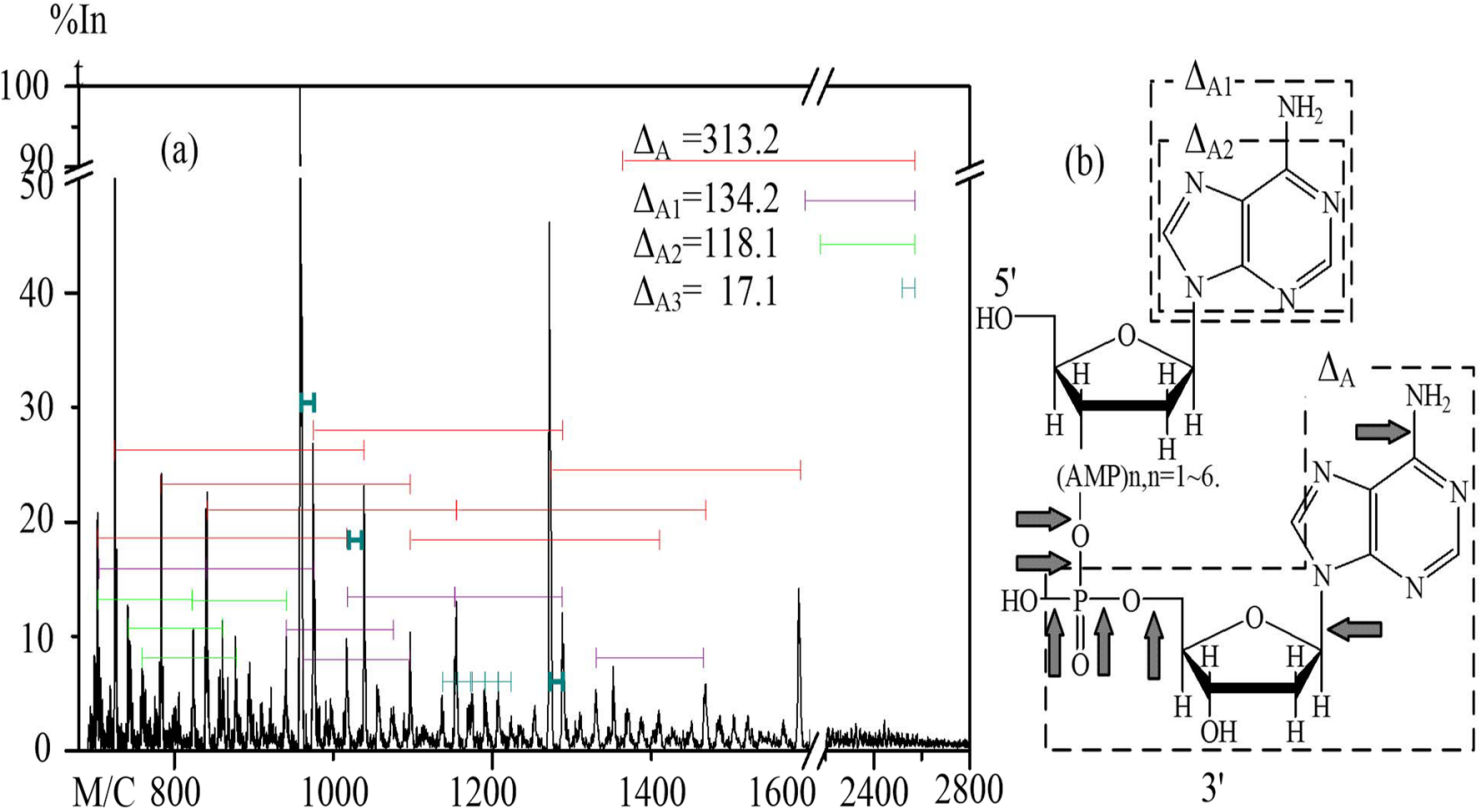
The MALDI-TOF mass spectrum of dA_8_ after a 4-min ARTP treatment. (**a**) The MALDI-TOF mass spectrum of dA_8_ showing differences in *m/z* values between various signals. (**b**) The structure of dA_8_. The arrows indicate bonds that would have been cleaved to generate fragments having the molecular weights assigned in the mass spectrum.

**Figure 5:**
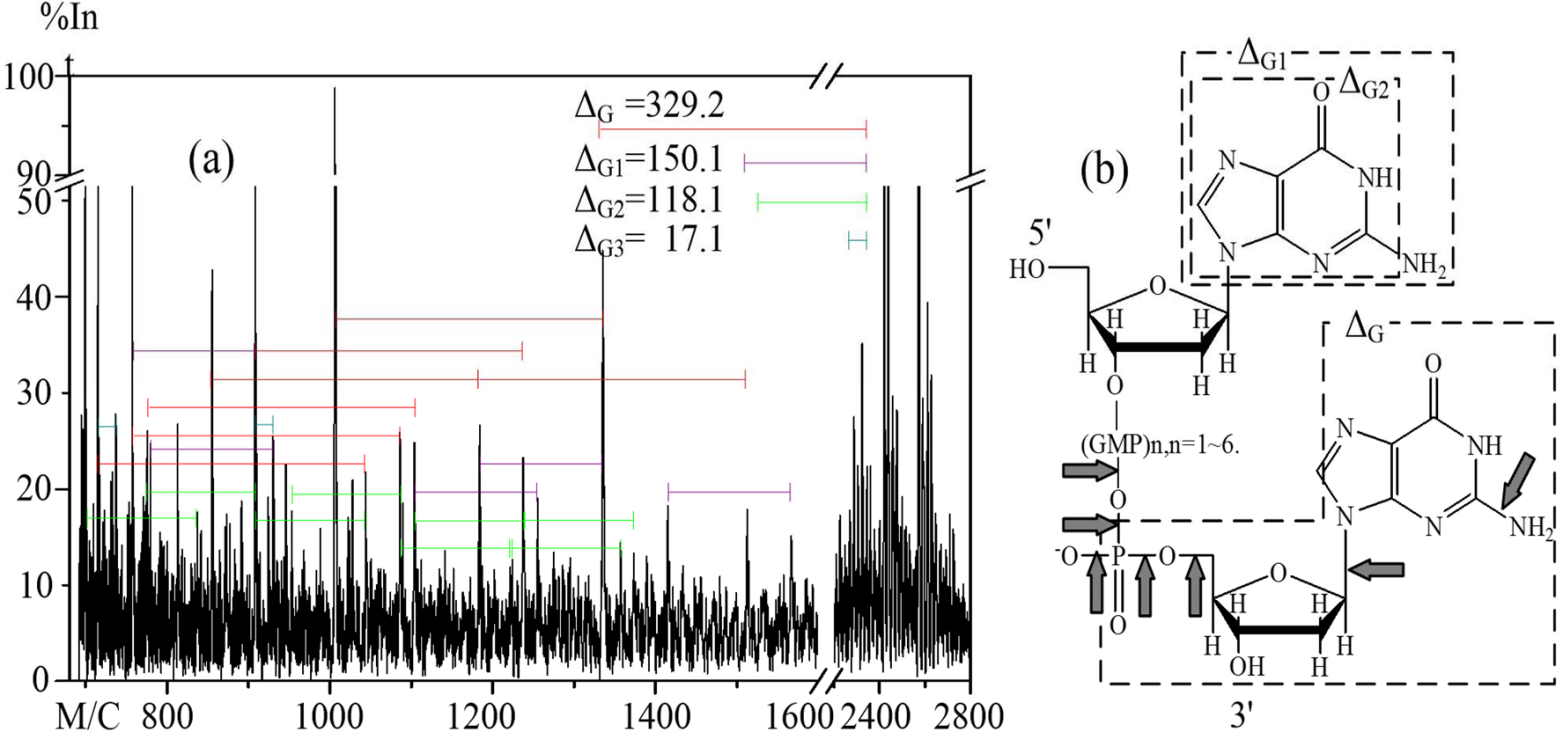
The MALDI-TOF mass spectrum of dG_8_ after a 4-min ARTP treatment. (**a**) MALDI-TOF mass spectrum of dG_8_ showing differences in *m/z* values between various signals. (**b**) The structure of dG_8_. The arrows indicate bonds that would have been cleaved to generate fragments having the molecular weights assigned in the mass spectrum.

**Figure 6:**
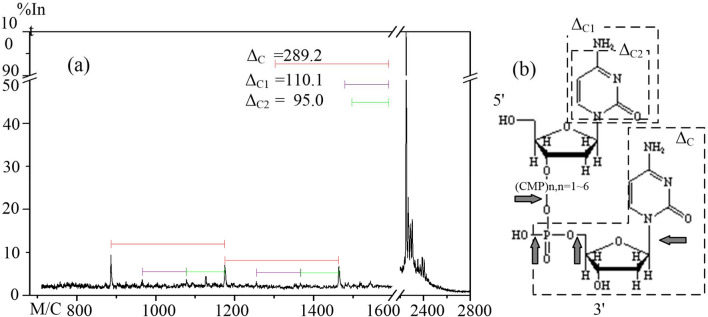
The MALDI-TOF mass spectrum of dC_8_ after a 4-min ARTP treatment. (**a**) MALDI-TOF mass spectrum of dC_8_ showing differences in *m/z *values between various signals. (**b**) The structure of dC_8_. The arrows indicate bonds that would have been cleaved to generate fragments having the molecular weights assigned in the mass spectrum.

**Figure 7:**
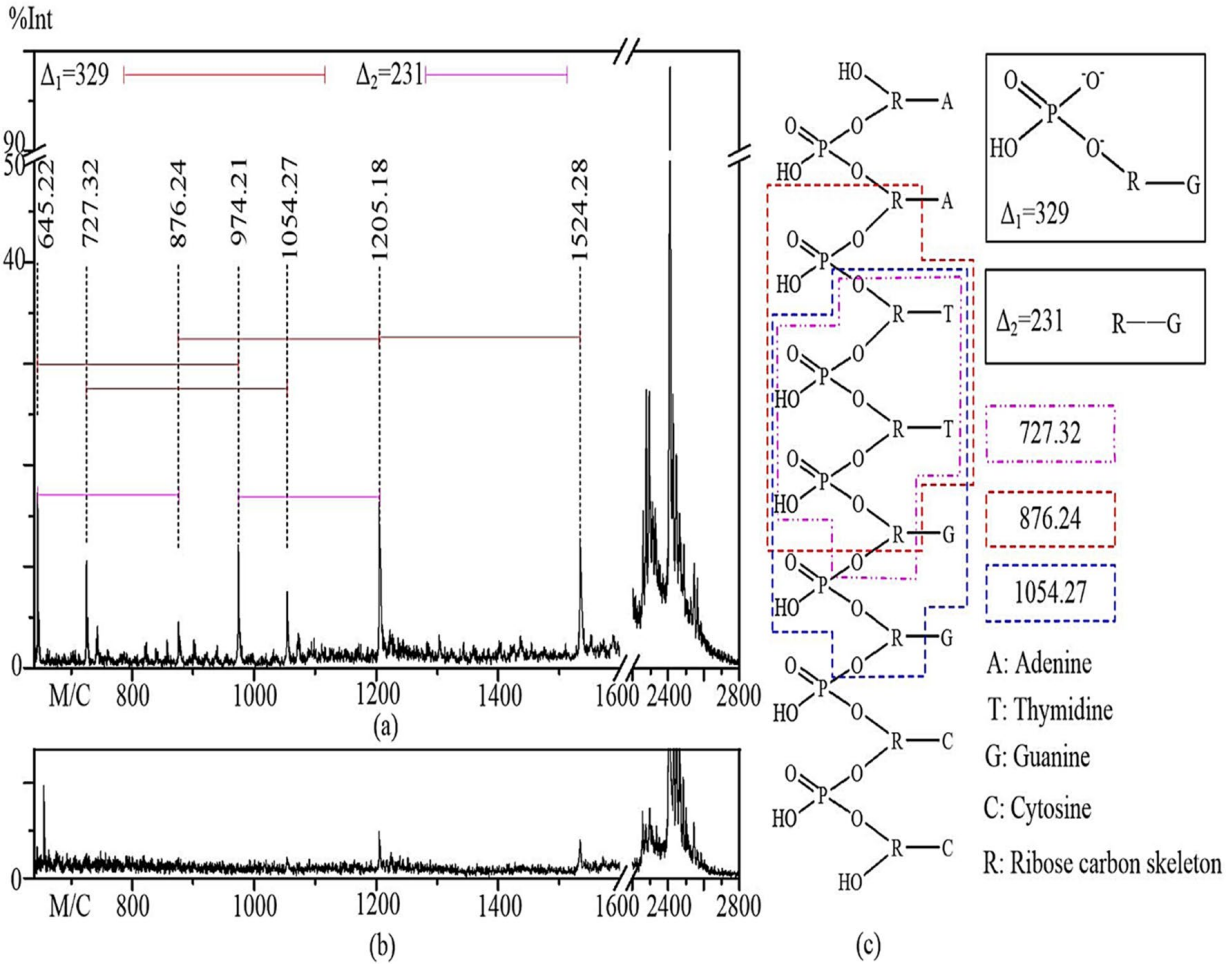
The MALDI-TOF mass spectrum of dA_2_dT_2_dG_2_dC_2_ after a 2-min ARTP treatment. (**a**) Mass spectrum of dA_2_dT_2_dG_2_dC_2_ after a 2-min plasma treatment. (**b**) Mass spectrum of untreated dA_2_dT_2_dG_2_dC_2_. (**c**) Assigned fragments produced by plasma treatment for the signals observed in the spectrum shown in (**a**).

The relationship between the molecular weight/mass of a molecule and the *m*/*z* values of oligonucleotides can be determined simply by reading the *m/z* values from the MALDI-TOF/MS spectra and solving the below equation. The chemical structure formulas of the fragments and the positions of the broken bonds can then be deduced, expressed as the following equation:

$$m/z = \, \left( {{\text{MW}} + {\text{nH}}^{ + } } \right)/{\text{n}}$$

where *m*/z is the mass-to-charge ratio marked on the abscissa of the spectrum, *MW* is the molecular mass of the molecule, *n* is the integer number of charges on the ions, and *H* is the mass of a proton = 1.008 Da^29^. The molecular weights of major peaks in the spectra (Figs. 4, 5, 6 and 7) was calculated using the above equation and the values of molecular mass difference between two peaks were named Δ_A_, Δ_A1_, Δ_A2_ and Δ_A3_ (Table 1).

**Table 1:** Fragments of oligonucleotides produced by treatment with plasma and identified by MALDI-TOF/MS (R, carbon skeleton of deoxyribose).

Oligonucleotide	Fragment	Molecular formula			Molecular mass (Da)		Chemical group
dA_8_	Δ_A_	C_10_ H_12_N_5_O_5_P–(OH)_2_			313.2		AMP
	Δ_A1_	C_5_H_4_N_5_–H			134.2		Adenine
	Δ_A2_	C_5_H_3_N_4_–NH_3_			118.2		Adenine minus its amino group
	Δ_A3_	NH_3_			17.1		Amino group
dG_8_	Δ_G_	C_10_H_12_N_5_ O_8_P–(O^–^)_2_			329.2		GMP
	Δ_G1_	C_5_H_4_N_5_O-H			150.1		Guanine
	Δ_G2_	C_5_H_2_N_4_O–NH_3_			118.1		Guanine minus its amino group
	Δ_G3_	NH_3_			17.1		Amino group
dC_8_	Δ_C_	C_9_ H_12_N_3_O_6_P–(OH)_2_			289.2		CMP
	Δ_C1_	C_4_H_4_N_3_O–H			110.1		Cytosine
	Δy	C_4_H_2_N_2_O–NH_3_			95.0		Cytosine minus its amino group
dT_8_	Stable to ARTP treatment						
dA_2_dT_2_dG_2_dC_2_	Δ_1_	HO_4_P^2–^-R-Guanine			329		GMP
	Δ_**2**_	R-Guanine			231		Carbon skeleton of deoxyribose plus a guanine

Notably, as mentioned above, dA_8_ was almost completely fragmented by a 4-min treatment with plasma. Accordingly, the chemical structures of the fragments and the bonds broken upon treatment with plasma were deduced based on the change in molecular weight (ΔMW) values between different fragments (Fig. 4a). The molecular weights and chemical formulas of the fragments corresponding to the main peaks in each spectrum were deduced and are listed in Table 1. Certain bonds in the dA_8_ skeleton, involving deoxyribose and/or a phosphate, were broken. Moreover, two fragments of dA_8_ with molecular weights of Δ_A1_ (134) and Δ_A2_ (118) (Fig. 4a) appear to correspond to those of adenine C_5_H_4_N_5_-H and adenine C_5_H_3_N_4_-NH_3_ (i.e., minus an amino group) (Table 1), respectively. These results suggested that the most easily broken bonds in dA_8_ were the C–N bond between C-1′ of the deoxyribose and N-9 of adenine, the N–C bond between the amino N and C of adenine, and the P-O bond between the phosphate and hydroxyl group (Fig. 4b).

The spectrum of dG_8_ was similar to that of dA_8_ after ARTP treatment, except for a residual amount of dG_8_ after a 4-min exposure to plasma (Figs. 3h and 5a). The differences between the molecular weights of the main signals were denoted Δ_G_, Δ_G1_, and Δ_G2_ and corresponded to GMP minus the two oxonium ions, an intact guanine, and a guanine without the amino group, respectively (Table 1). As was the case with dA_8_ (Fig. 4b), the C–N bond between C-1′ and N-9, the N–C bond between the amino N and C, and the P-O bond between the phosphorous and hydroxyl group were the most easily cleaved. Both adenine and guanine are planar purine bases, and the structures of these bases might inhibit interaction with their deoxyribose and phosphate components and show the more stability to the connection^30–32^ (Fig. 5b).

As shown in Fig. 6, the molecular structure of dC_8_, according to its mass spectrum, was more stable to ARTP treatment than was dA_8_ or dG_8_. After dC_8_ was treated with ARTP for 4 min, three weak peaks were detected at *m*/*z* values of 886.4, 1,175.2, and 1,464.3 (Figs. 3i and 6a). The Δ*m*/*z* value (Δ_C_) that corresponds to the difference between adjacent peaks was 289.2, which is that of dCMP (Fig. 6a). In addition, two other weak signals were detected at *m*/*z* values of 972.2 and 1,082.3 (Fig. 6a). The Δ*m*/*z* values were 110.1 (Δ_C1_) and 95.0 (Δ_C2_), which agree with the theoretical *m*/*z* values for cytosine and phosphate, respectively (Fig. 6b and Table 1). The phosphodiester fragments between the phosphorous and the hydroxyl group in the deoxyribose of dC_8_ appeared to be readily cleaved by ARTP treatment (Fig. 6b).

Finally, when the ARTP treatment time was 4 min, a weak signal was detected that could not be assigned to a fragment of dT_8_ (Fig. 3p). The appearance of this peak suggested that a small number of dT_8_ molecules were cleaved into small fragments by ARTP treatment (Fig. 3p).

The aforementioned observations, i.e., that the stability of each of dC_8_ and dT_8_, which contain pyrimidine bases, was higher than the stability of dA_8_ or dG_8_, which contain purine bases, suggested that the relatively larger purine bases have ribose-phosphate backbones that are relatively more exposed to the environment as a consequence of linearization of the chains compared with the pyrimidine bases of dC_8_ and dT_8_; hence, this aspect made the backbones of dA_8_ and dG_8_ more susceptible to ARTP-induced cleavage.

To begin to characterize the ARTP-induced fragmentation products of single-stranded hetero- oligonucleotide chains, the synthetic oligonucleotide, dA_2_dT_2_dG_2_dC_2_, was subjected to the treatment with ARTP for 2 min as described for the homo-oligonucleotide chains, and the resulting fragments were also identified by MALDI-TOF/MS (Fig. 7). Differences between pairs of signals shown in Fig. 7a were 329 (Δ_1_) and 231 (Δ_2_), which agree with the molecular weight of GMP and that of a guanine attached to the carbon skeleton of deoxyribose (Fig. 5a), respectively. The speculative chemical structures of assigned fragments (Fig. 7c) produced by plasma treatment for the peak signals with *m*/*z* values of 727.22, 876.24, and 1,054.27 observed in the spectrum in Fig. 7a, respectively. The spectrum of nontreated dA_2_dT_2_dG_2_dC_2_ by ARTP is shown in Fig. 7b and also contains signals with *m*/*z* values of 727.32, 876.24, and 1,054.27 corresponding to Fig. 7a, respectively.

The aforementioned results indicated that the phosphodiester fragments of the mononucleotides and oligonucleotides were easily broken by ARTP. In addition, the N–C bonds between the bases and the deoxyribose moieties of dA_8_ and dG_8_ were also easily broken to generate adenine and guanine, respectively. Compared with the cleavage of adenine and guanine from their homonucleotide chains, cleavage of thymine and cytosine was more difficult. The interpretation of these results was similar to that for dC_8_ and dT_8_ as discussed above. It was thus concluded that the stabilities of single-stranded oligonucleotides exposed to ARTP were strongly influenced by the bases present.

### Molecular dynamic simulation of the oligonucleotides

Considering that potential energy level can reflect the stability of a matter to a certain extent, we used the molecular dynamic simulation with Discovery Studio v2.5 in terms of energy minimization of the oligonucleotides to determine their stability. Taking that the concentration of oligonucleotides we used in ARTP treatment was 200 mM, which was equivalent to that one oligonucleotide was solvated in around 300 water molecules into consideration, we constructed a cubic box with about 390 water molecules for solvation of a nucleotide for the molecular simulation (Fig. S3, Fig. S4). As shown in Table 2, the average potential energy for the respective dA_8_, dT_8_, dG_8_, dC_8_ and dA_2_dT_2_dG_2_dC_2_ used in the experiment were − 16.326, − 16.556, − 16.268, − 16.362 and − 16.202 kcal/(mol/molecule), respectively. The average potential energy of the oligonucleotides was dT_8_ < dC_8_ < dA_8_ < dG_8_ < dA_2_dT_2_dG_2_dC_2,_ indicating that the turn of the oligonucleotide stability was dT_8_ > dC_8_ > dA_8_ > dG_8_ > dA_2_dT_2_dG_2_dC_2._ This is consistent with our experimental results described above.

**Table 2:** Potential Energy after energy minimization during the simulation.

	dA_8_	dT_8_	dG_8_	dC_8_	dA_2_dT_2_dG_2_dC_2_
Potential energy (kcal/mol)	− 6,465	− 6,308	− 6,491	− 6,365	− 6,173
Total molecules in a cubic box (Number of water molecules and oligonucleotides)	396	381	399	389	381
Average potential energy (kcal/mol/molecule)	− 16.326	− 16.556	− 16.268	− 16.362	− 16.202

## Discussion

Mutation is a hereditable change in the structure of DNA of an organism altered by physical or chemical mutagens either directly or indirectly. However, the main difficulty for an organism mutation is how to know the mutagenic effectiveness and in vivo variation in genomic DNA genomes with particular mutagens^32,33^. For example, ultraviolet radiation (UV) is one of widely used physical mutagens capable of inducing mutagenic DNA lesions^34,35^. The possible mutagenic mechanisms caused by UV-induced DNA damage were pyrimidine dimers such as cyclobutene-pyrimidine dimers, 6–4 photoproducts, and their Dewar valence isomers as well as DNA strand breaks by interfering the genome integrity^34–36^. By far, many mutagenesis approaches to damaging DNA to create mutations using physical mutagens (e.g., ^60^Co γ-rays, X-rays, UV light, radiofrequency radiation, etc.)^37,38^ have been studied and applied. Some of possible mutagenic mechanisms caused by physical mutagens have been elucidated^34–40^.

More and more experimental results have also showed that the ARTP as a new kind of physical mutation tool has a bright prospect for the genome mutation of the organisms with rapid mutation, high diversity of mutants and high safety^1,4^. Up to now, ARTP as a novel powerful mutagenesis method has been successfully used to mutation breeding of more than 110 kinds of microorganisms and plants as well animals^19–22,25^. Generally, mutagenesis of organisms is a complex process, dependent on the cellular DNA damage strength and the subsequent repair system of damaged DNA, which allows mutation to occur. Our previous study demonstrated that compared with UV and chemical mutagens, ARTP showed highest DNA damage strength per living cells and mutation rate per generation^26^. Thus, how to obtain the direct molecular evidence for the DNA base pair damage by ARTP irradiation is needed to understand the ARTP mutagenesis mechanisms.

In this study, the NMR spectroscopy and MALDI-TOF/MS employed to detect artificial chemical building blocks of DNA (mononucleotides and oligonucleotides) in vitro treated by ARTP, we successfully analyzed and deduced the mononucleotide and oligonucleotide products, respectively. Since this study aimed to explore the direct damage process of ARTP to oligonucleotides and no publication could be found as reference to design the experiment, we employed ARTP to treat the hetero-nucleotides with 2 residues which could be synthesized simply to predict what would happen in the structure of the simplified oligonucleotides. We found that altered ^31^P triphosphate signals, and ^1^H deoxyribose and base signals in the corresponding spectra of the mononucleotides indicated that treatment with plasma affected the structures of the mononucleotides. The ^31^P-NMR spectra directly indicated that the phosphate bonds of the mononucleotides were broken upon treatment with plasma. With different damaged-DNA results in vivo, which in clustered DNA damage of severity greater than simple double-strand breaks caused by ionizing radiation^37^, ARTP treatment broke the artificial oligo-nucleotides in vitro into small fragments, as evidenced by MALDI-TOF/MS. The purines of dA_8_ and dG_8_, and the purines minus their amino groups, were cleaved by treatment with plasma, and CMP, cytosine, and phosphate were released from dC_8_. Compared with the products found for dA_8_, dG_8_, and dC_8_, the structural stability of thymidine was greater than that of the other bases because we found that dT_8_ was hardly cleaved under the same experimental conditions. The structural alterations (stability) of the oligonucleotides in vitro induced by ARTP appeared to be attributable primarily to the base.

To further understand the stability of the synthesized oligonucleotides, molecular dynamic simulation was performed to generate the three dimensional pharmacophore model for simulation of the single-stranded homo- and hetero-oligonucleotides used in this study (Figs. S3 and S4). Since the potential energy level obtained can reflect the stability of a matter, we used energy minimization to determine the stability of the oligonucleotides used in this study. The order of the stability was dT_8_ > dC_8_ > dA_8_ > dG_8_ > dA_2_dT_2_dG_2_dC_2_ (Table 2), which was consistent with our experimental results by ARTP treatment of the respective oligonucleotides.

The above experimental results showed that ARTP could diversely damage DNA base molecules, implying the effective mutation capability for many kinds of organisms as mentioned in Introduction. However, by far we still hardly link the current deduced results to organism mutation mechanism by ARTP in vivo, since for in vivo mutation, in addition to the direct breakage of DNA at the first step, the complicated repair system for the damaged DNA during cell survival growth is another important factor, which make the mutation process analysis complicated. Recently, whole-genome sequencing also revealed the in vivo mutation features of Japanese flounder, a sea fish of *Paralichthys olivaceus* caused by ARTP mutagenesis, which showed multiple SNP and InDel mutation sites and the average mutation rate was up to 0.064% at the genome level, which was 2–3 order higher than the traditional mutagenesis methods^23^. According to bioinformatics analysis, the higher mutation rates were found in many key genes associated with cellular process, metabolic process, biological regulation processes, cell components, binding function and catalytic activity in mutated species of *P. olivaceus*^23^. By multiplex ARTP mutagenesis of *Zymomonas mobilis*, a bacterial strain for ethanol production, a mutant with improved tolerance to both high acetic acid concentration up to 8 g/L and low pH down to pH 3.5 was obtained, which showed enhanced growth and ethanol production under both sterilized/unsterilized conditions at pH 4.0 or 3.5^24^. By genomic resequencing analysis of the tolerant mutant, eleven single nucleic variations (SNVs) found to be likely related to acetic acid and low pH tolerance, and these SNVs were interestingly distributed between genes of ZMO0952 and ZMO0956, ZMO0152 and ZMO0153, and ZMO0373 and ZMO0374 in the genome, indicating the uniqueness of ARTP mutagenesis for strain improvement^24^. Taken together with these examples of ARTP mutagenesis, the present study showed the first-hand evidence for the DNA base damage pattern by ARTP irradiation. More studies on the comparative bioinformatic analysis for the selected different mutants from one same starting organism by ARTP mutagenesis will be needed further to provide deep insights into comprehensive molecular mutation mechanism of the mutated organisms by genotype and phenotype association.

## Conclusions

In this study, we found that various chemical structural changes to the artificial mononucleotides and oligonucleotides that may account mutagenesis for the wide and successful applications reported for ARTP-induced mutation breeding of diverse microbes and plants as well animals caused by ARTP treatment. The results of ^31^P-and ^1^H**-**NMR for the plasma-treated and untreated mononucleotides indicated that the chemical natures of the mononucleotides determined their stability to irradiation with ARTP and dATP was less stable than were the other mononucleotides. The oligonucleotides after treatment with ARTP have been cleaved bonds and broken into small fragments as detected by mass spectrometry. The stabilities of the oligonucleotides differed to ARTP irradiation, with dT_8_ being the most stable and was more beneficial to stabilizing single-stranded oligonucleotide structures compared to the other base groups (A, G, and C). The different structure stability of the oligonucleotides to ARTP irradiation was also confirmed by the molecular dynamic simulation. The order of average potential energy of the oligonucleotides was dT_8_ > dC_8_ > dA_8_ > dG_8_ > dA_2_dT_2_dG_2_dC_2_. Therefore, these changes of chemical building blocks of DNA also provide a basic insight into the mechanism understanding of ARTP mutagenesis, a tool that has been widely applied for mutation breeding of different organisms. And also the ARTP as a powerful mutagenesis method will feasibly contribute to the progress of a comprehensive study on an organism mutation and evolution.

## Materials and methods

### Sample preparations

The mononucleotides dATP, dGTP, dCTP, and dTTP were purchased from Takara Co., Ltd (Dalian, China). The single-stranded oligonucleotides dA_8_, dT_8_, dG_8_, dC_8_, and dA_2_dT_2_dG_2_dC_2_ were synthesized and purified with HPLC by Invitrogen Life Technologies Ltd (Shanghai, China). The mononucleotides and oligonucleotides were dissolved in distilled, nuclease-free water at concentrations of 100 and 200 mM, respectively, and for each sample, 5µL was subjected to ARTP treatment.

### ARTP jet instrumentation

The equipment used to generate the helium radio-frequency ARTP jet consists of a 13.56-MHz power supply, a co-axial-type plasma generator, a helium gas supply and control subsystem, and a stainless-steel sample plate that can be moved smoothly in the vertical direction to adjust the stand-off distance between the plasma torch nozzle exit and the sample plate^26,27^ (Fig. 1). The experimental parameters were as follows: The radio-frequency power input was 120 W, the stand-off distance was 2.0 mm, and the plasma jet temperature 2.0 mm downstream of the plasma torch nozzle exit was < 40 ºC (Fig. 1).

### NMR spectroscopy

After repeated lyophilization with D_2_O solution, the freeze-dried samples were dissolved in 0.5 mL of 99% D_2_O (Sigma) for NMR studies. ^31^P- and ^1^H-NMR spectroscopy were performed according to the reference with slight modification^41,42^. Briefly, for ^31^P- NMR spectroscopy, the solution-state 31P NMR spectra were acquired at 24 °C using a JEOL ECA 600 spectrometer (JEOL Ltd., Japan) operating at 243 MHz with a 45° pulse width (5.2 µs pulse), an acquisition time of 0.77 s (50 ppm window centered at 0 ppm), a delay time of 2.0 s. The chemical shifts were determined in relation to external 85% H_3_PO_4_ (at δ = 0). All of the spectral results were processed with the NMR Utility Transform Software (NUTS) for Windows (Acorn NMR, Livermore, CA). Signals for P compounds or functional groups were determined according to literature^43^.

For ^1^H-NMR spectroscopy, the ^1^H spectra were recorded on a JOEL JNM ECA-600 spectrometer operating at 600.17 MHz at 25 °C with D_2_O as the internal lock. All ^1^H NMR spectra were acquired with inverse detection probe. The parameters used were 32 sans, 16,384 points, spectral width of 15 ppm, 90 °C pulse length of 5.2 µs and relaxation delay of 2 s. To suppress the residual water signals, a pre-saturation sequence was used at the water frequency during the recycle delay. ^1^H NMR spectra free induction decays were zero filled to 64 K before Fourier transformation, and then were transformed with a line broadening factor of 0.3. Transformed ^1^H spectra were manually phased, baseline corrected and calibrated to TMSP at 0.0 ppm with Mnova (version 8.1.2, Bruker). 2D NMR spectra including ^1^H J-resolved spectroscopy (JRES), ^1^H-^1^ H correlation spectroscopy (^1^H-^1^HCOSY), ^1^H-^1^H total correlation spectroscopy (^1^H-^1^H TOCSY), heteronuclear single quantum coherence spectroscopy (HSQC) and heteronuclear multiple bond correlation (HMBC) were also recorded on the 600 MHz JEOL spectrometer with parameters.

### MALDI-TOF/MS

MALDI-TOF mass spectra were acquired on a Waters MALDI micro MX instrument to detect the product-ion m/z values obtained for the plasma-treated and untreated oligonucleotides. The matrix was composed of 0.1% (w/v) 2, 5-dihydroxybenzoic acid in a 50:50 (v/v) mixture of trifluoroacetic acid and acetonitrile. The matrix and each sample solution, 0.5 μL each, were mixed, and 0.8 μL of each mixture was individually and rapidly dropped into the sample well of the mass spectrometer and then dried at room temperature. The mass spectra were obtained in the negative mode on a time-of-flight Microflex mass spectrometer (Bruker), and the N2-laser excitation wavelength was 337 nm. Spectra were calibrated using reference oligonucleotides of known masses.

### Methods of energy minimization

Discovery Studio v2.5 (DS, www.accelrys.com) was used to generate the three dimensional pharmacophore model for the single-stranded homo- and hetero-oligonucleotides. All minimizations were based on CHARMM-27 force field. The oligonucleotides water models were used in order to create the aqueous environment. In DS, oligonucleotides were solvated in a cubic box with about 390 water molecules. Particles Mesh Ewald (PME) electrostatic and periodic boundary conditions were applied in all directions. The system was then subjected to a smart minimizer algorithm for minimization with 10,000 steps. Average Potential Energy of was defined as following equation: Average Potential Energy (kcal/mol/molecule) = Potential Energy/mol/molecule (including number of water molecules and oligonucleotide residues in one cubic box).

## Supplementary information

Supplementary information.

## Data Availability

No datasets were generated or analysed during the current study.
