# Supplementary material for: Insights into the molecular-level effects of atmospheric and room-temperature plasma on mononucleotides and single-stranded homo- and hetero-oligonucleotides
Source: Sci Rep. 2020 Aug 31;10:14298. doi: 10.1038/s41598-020-71152-1 (PMC7459345; doi:10.1038/s41598-020-71152-1)
Supplement: Supplementary file 1 — Supplementary information. [file 41598_2020_71152_MOESM1_ESM.pdf]

Supporting information

Insights into the molecular-level effects of atmospheric and room-temperature plasma on mononucleotides and single-stranded homo- and hetero-oligonucleotides

Liyan Wang[1, 2, 3†], Hongxin Zhao[4†], Dong He[1], Yinan Wu[1], Lihua Jin[5], Guo Li[6], Nan Su[1], Heping Li[6]* & Xin-Hui Xing[1, 7, 8]*

Figure S1

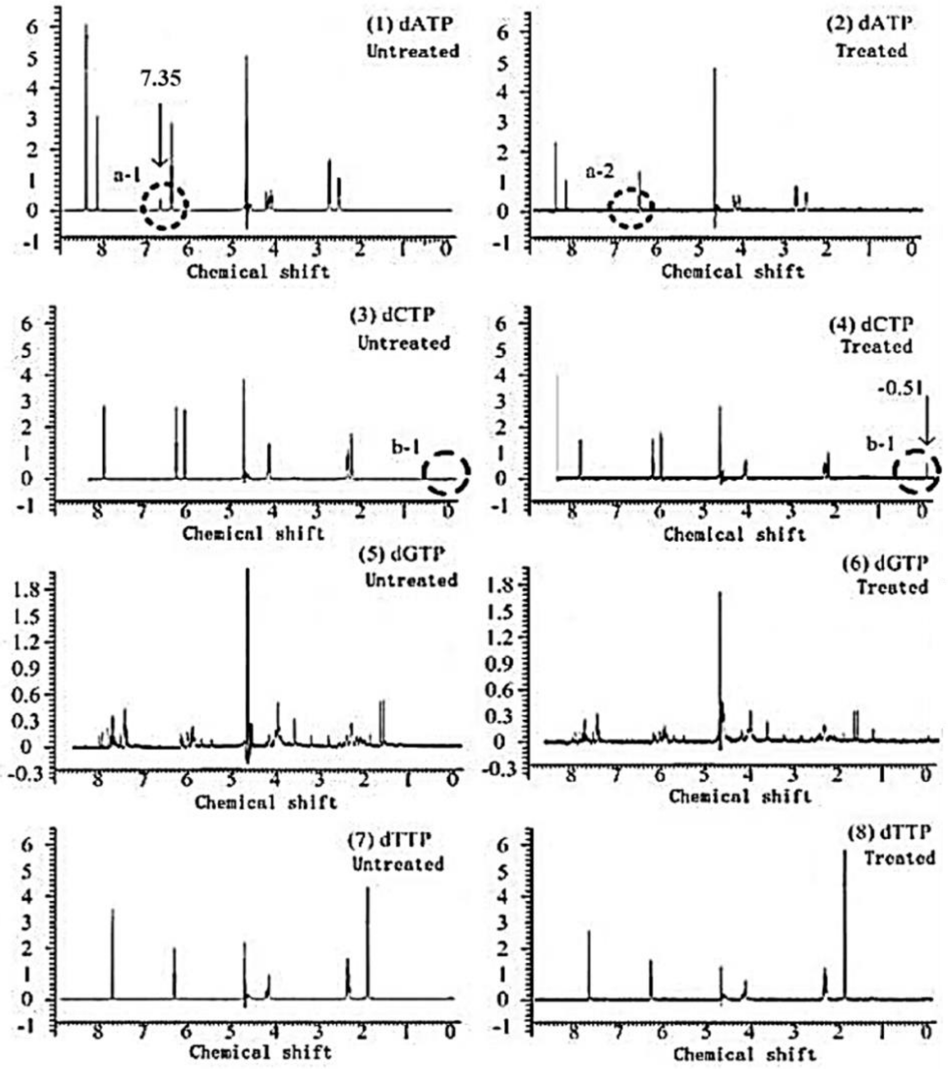

Fig. S1. 1H NMR spectra of the four different tri-phosphate mononucleotides.

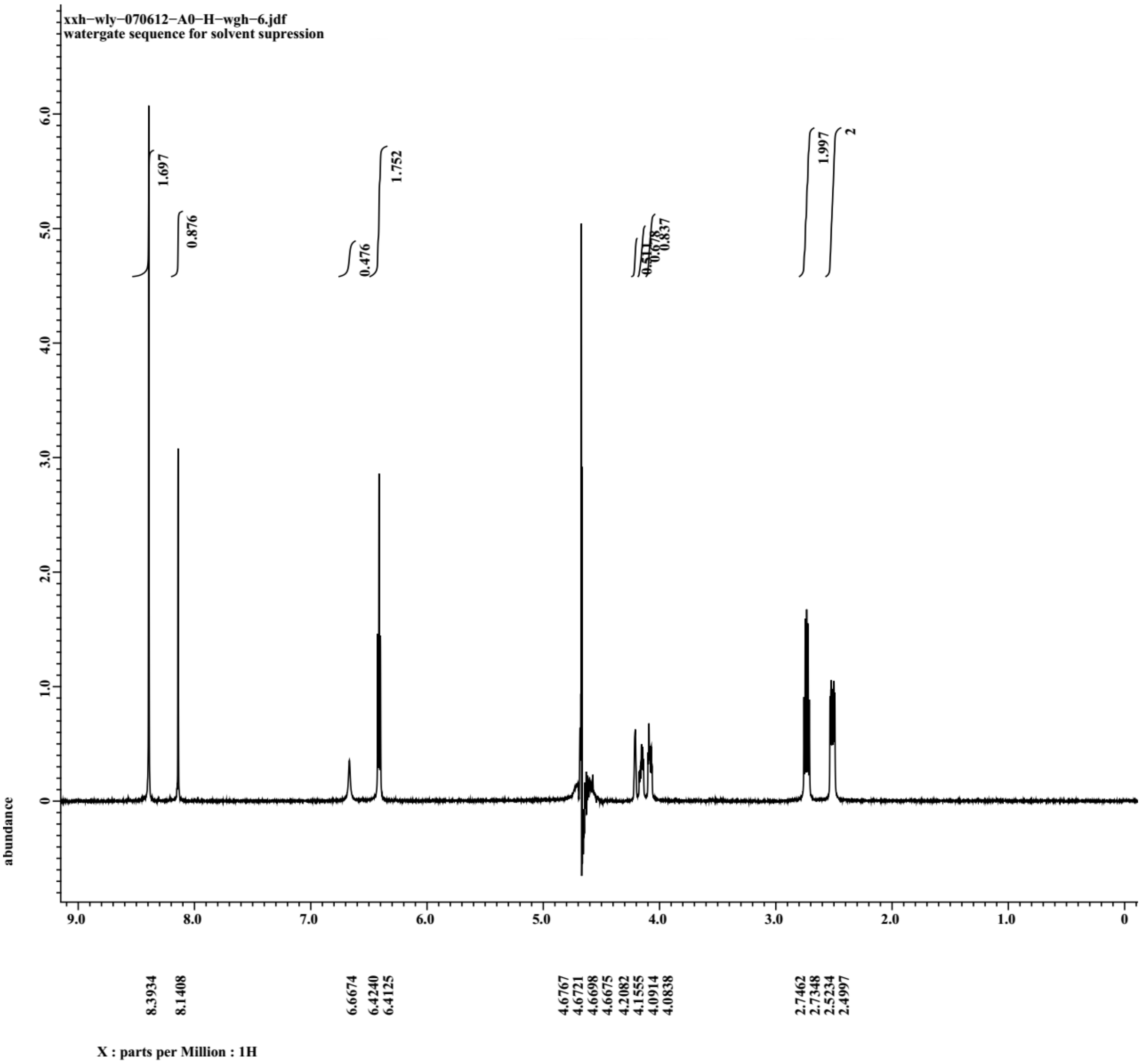

Raw data of Fig. S1. (1) dATP untreated

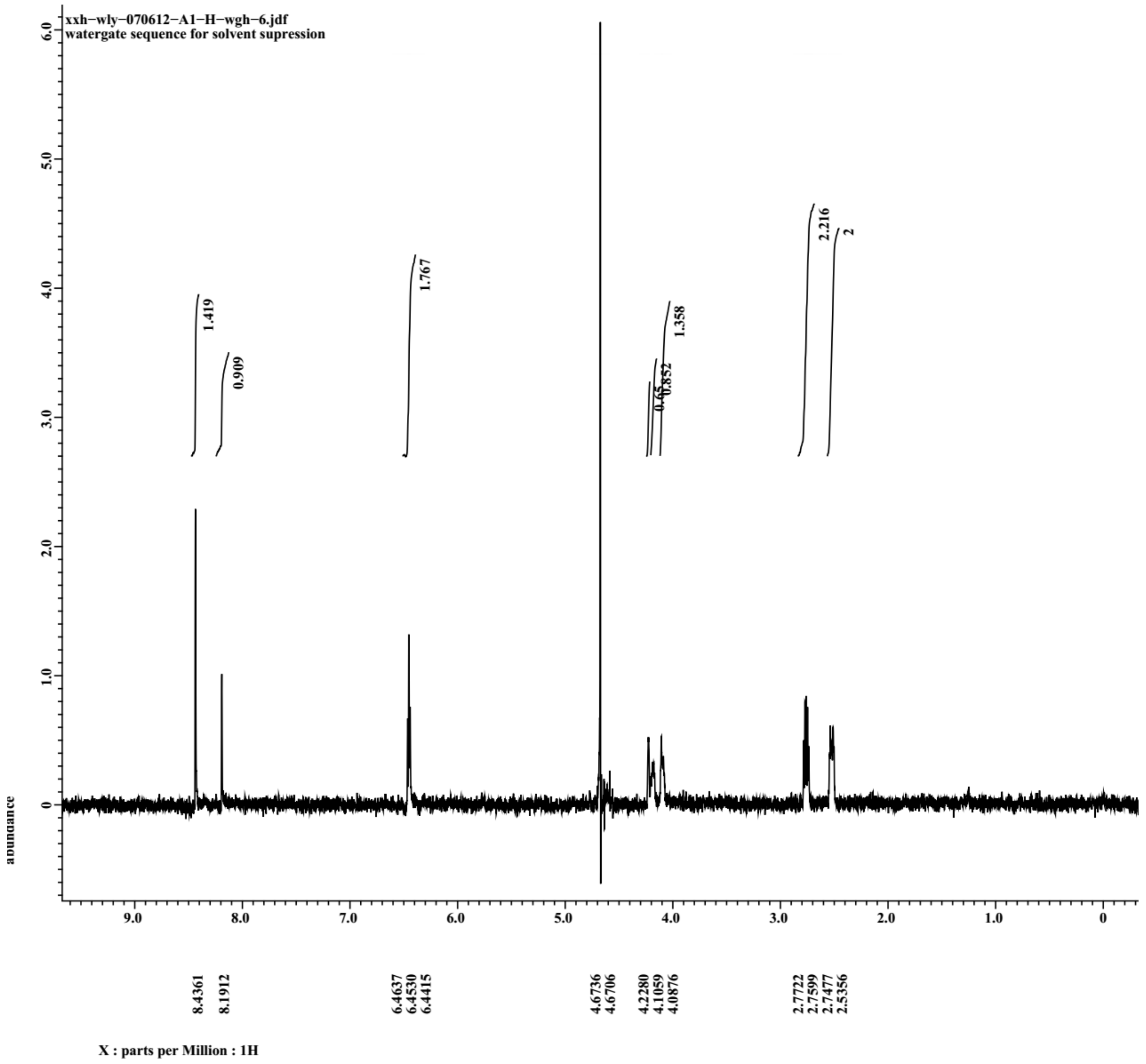

Raw data of Fig. S1. (2) dATP treated

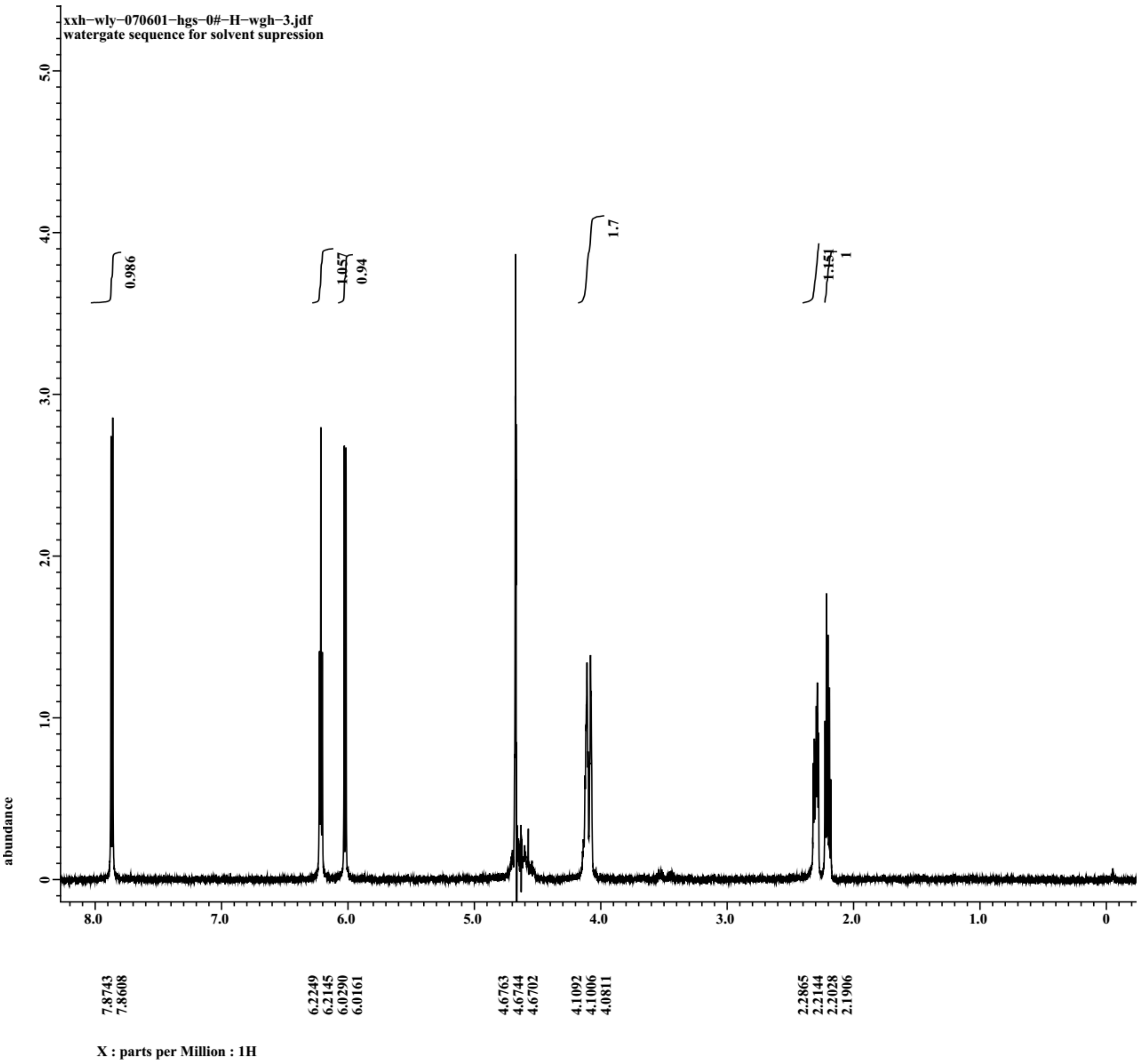

Raw data of Fig. S1. (3) dCTP untreated

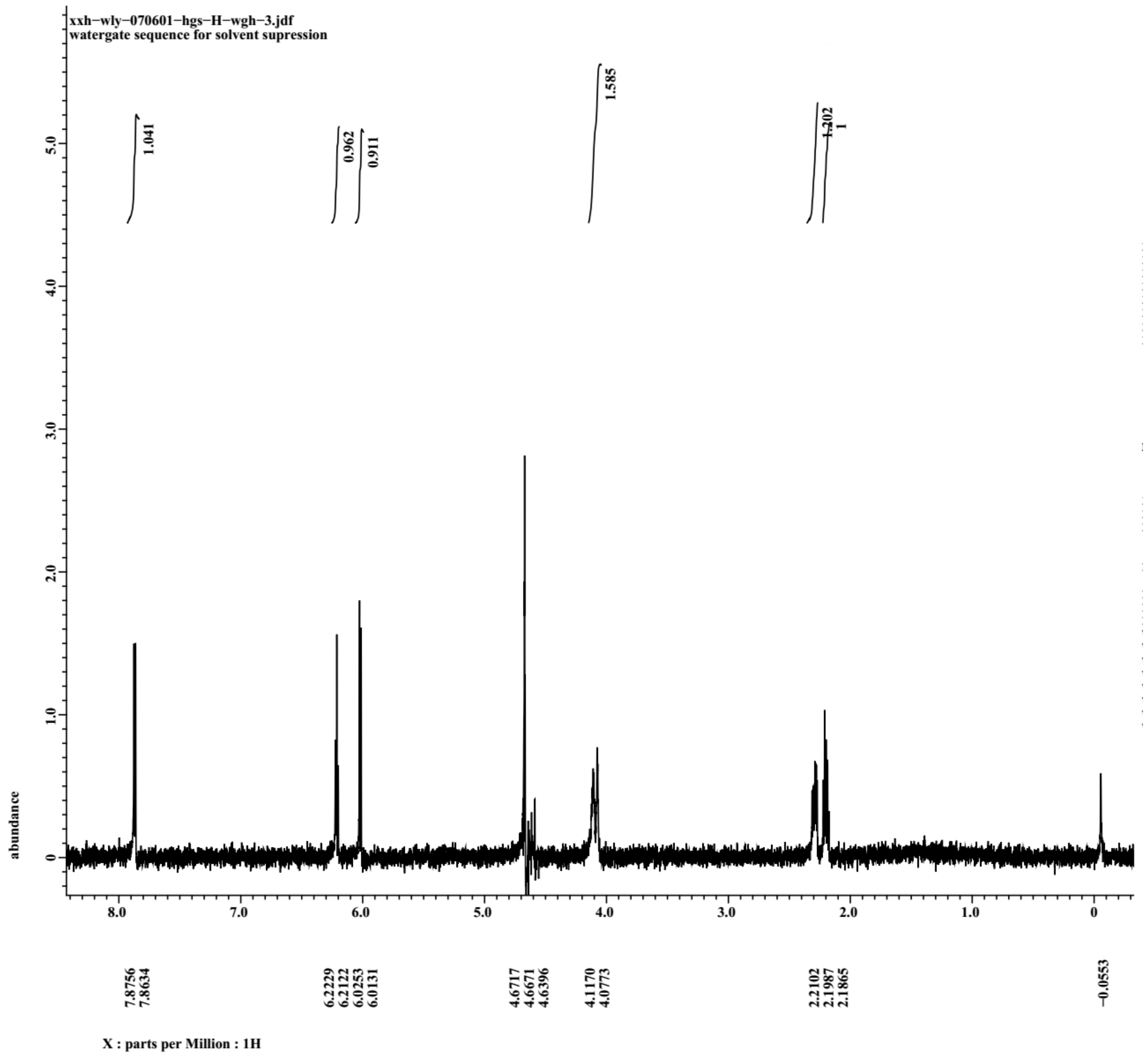

Raw data of Fig. S1. (4) dCTP treated

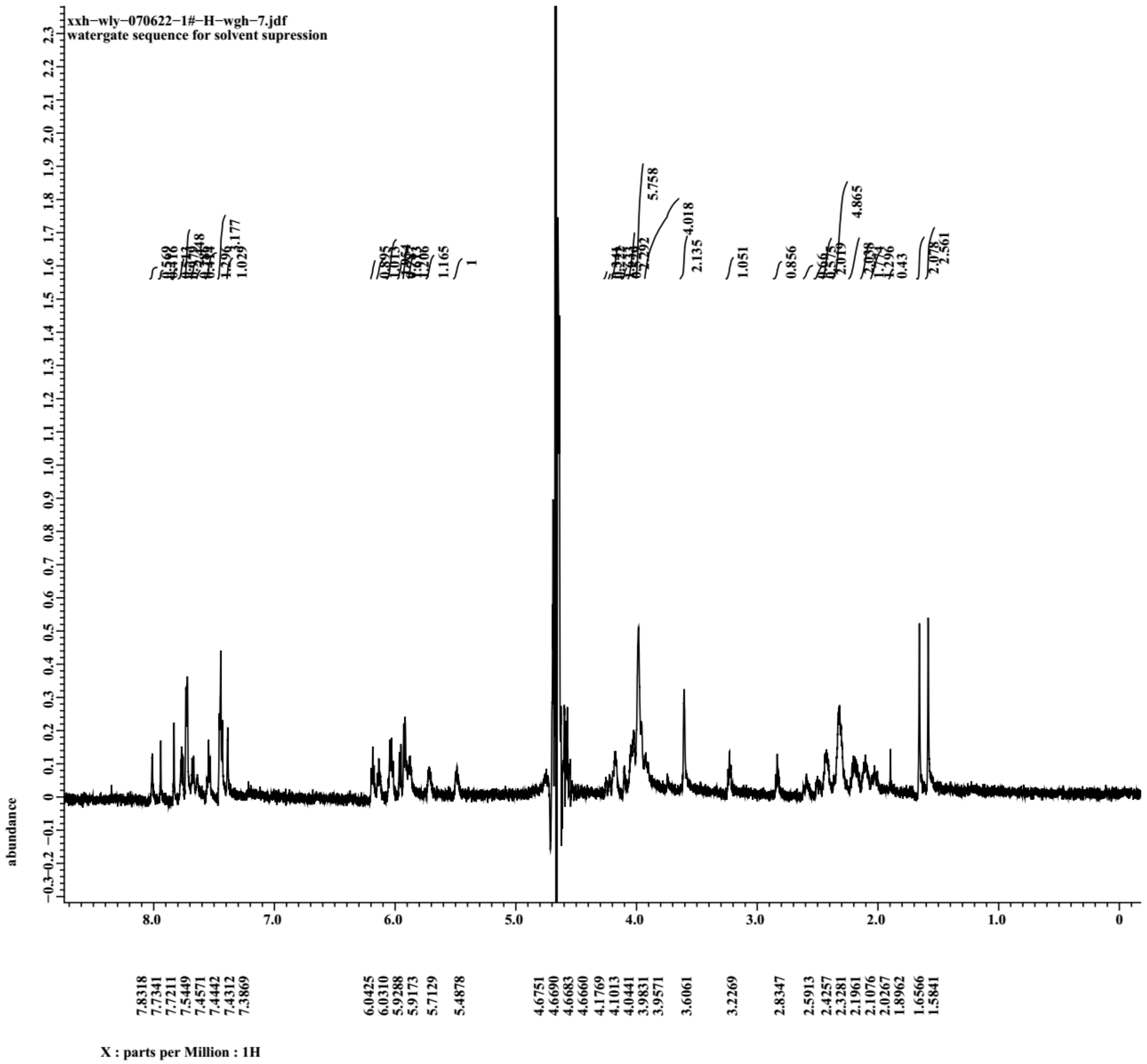

Raw data of Fig. S1. (5) dGTP untreated

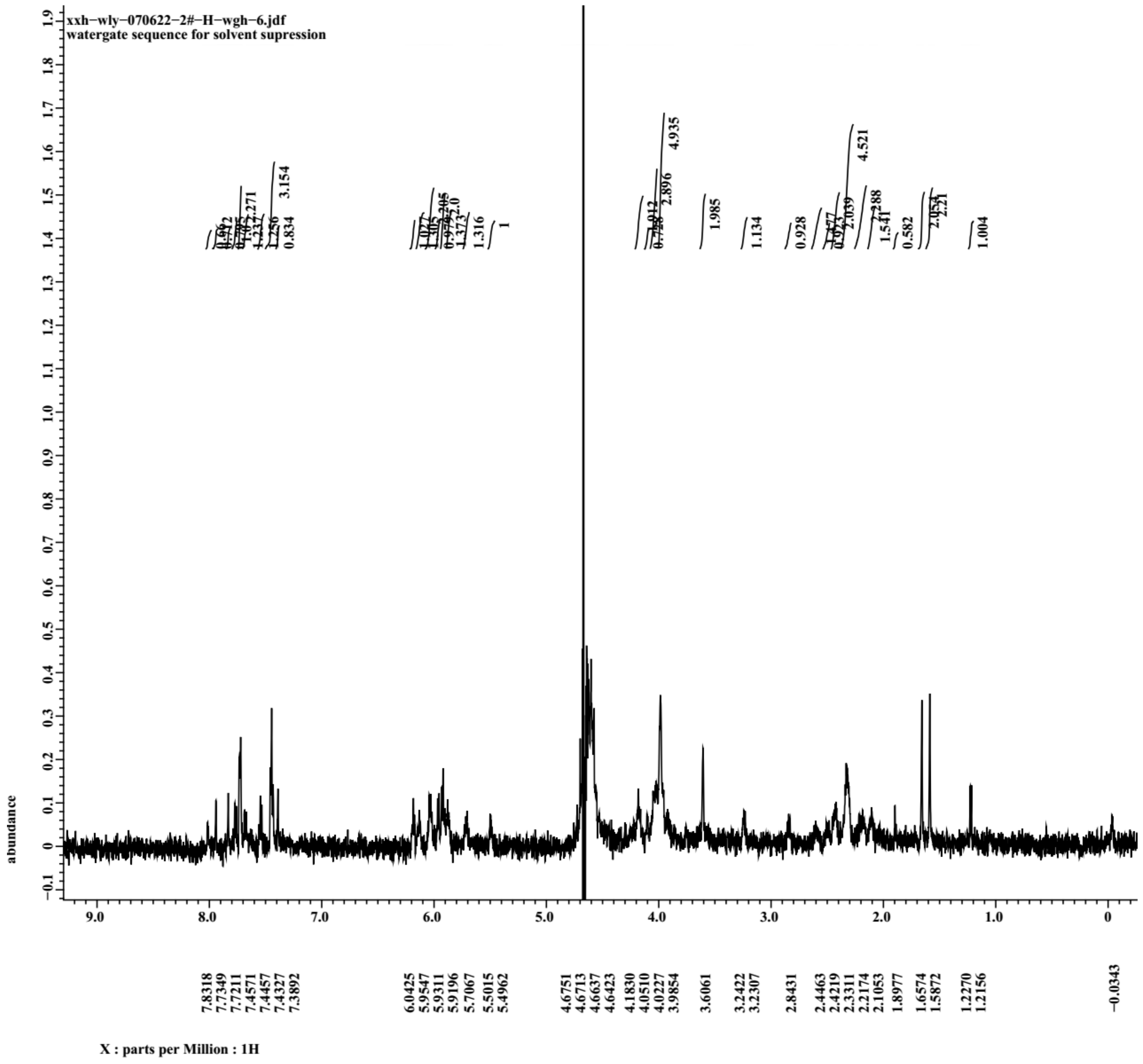

Raw data of Fig. S1. (6) dGTP treated

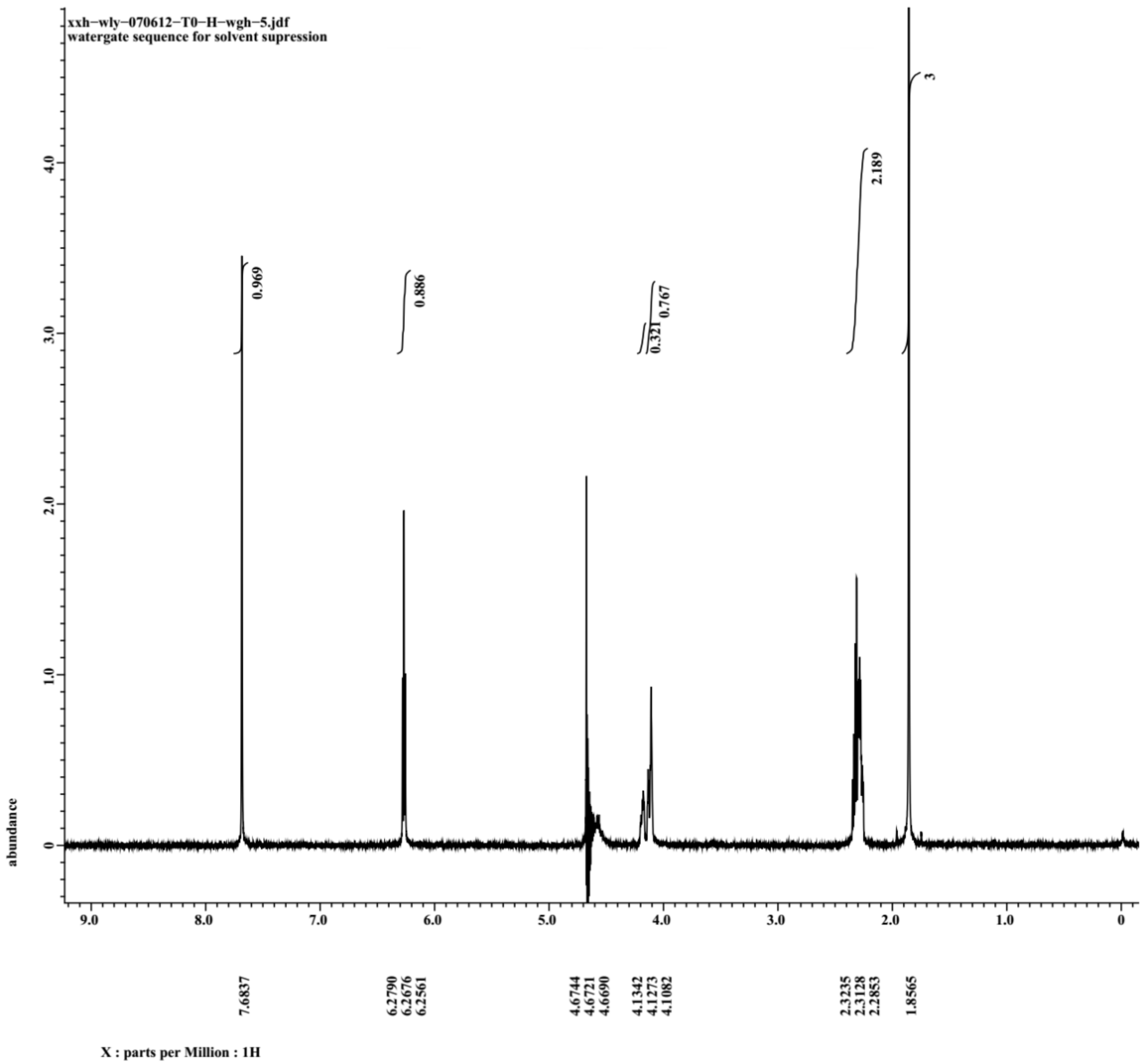

Raw data of Fig. S1. (7) dTTP untreated

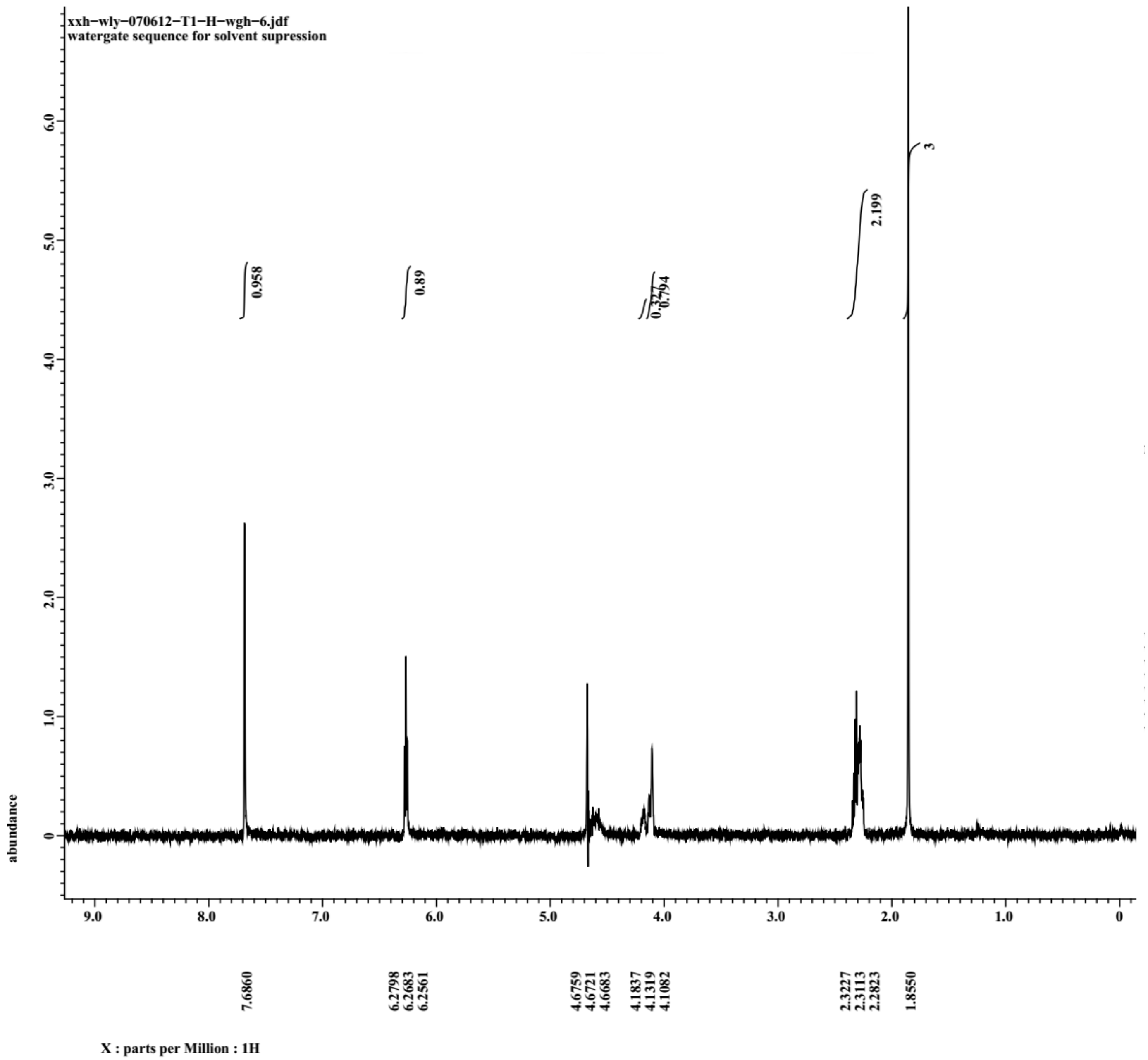

Raw data of Fig. S1. (8) dTTP untreated

Figure S2

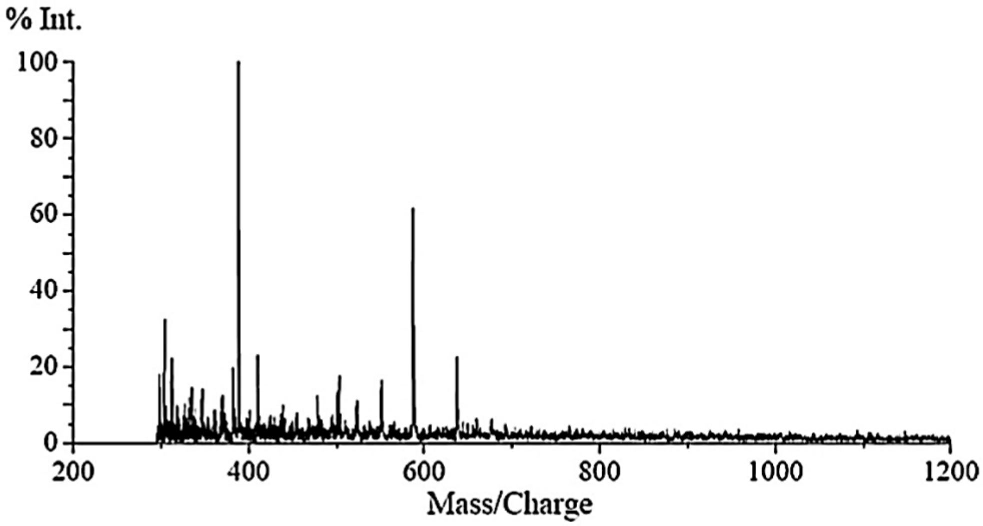

Fig. S2. The Maldi-TOF MS spectrums of the distilled water.

Figure S3

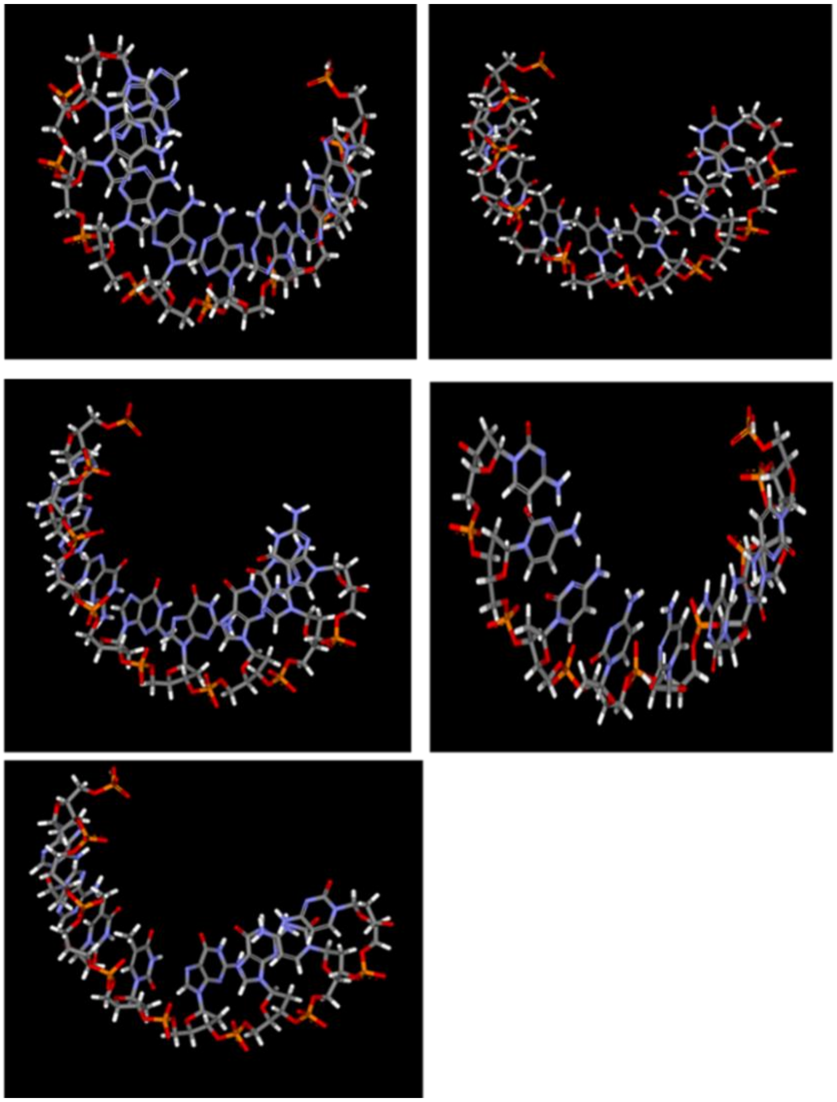

dA_8 dT_8

dG_8 dC_8

$dA_2dT_2dG_2dC_2$

Fig. S3. Molecular modeling of oligonucleotides without water molecules. Blue, blue, white and red represent carbon, nitrogen, hydrogen and oxygen, respectively.

Figure S4

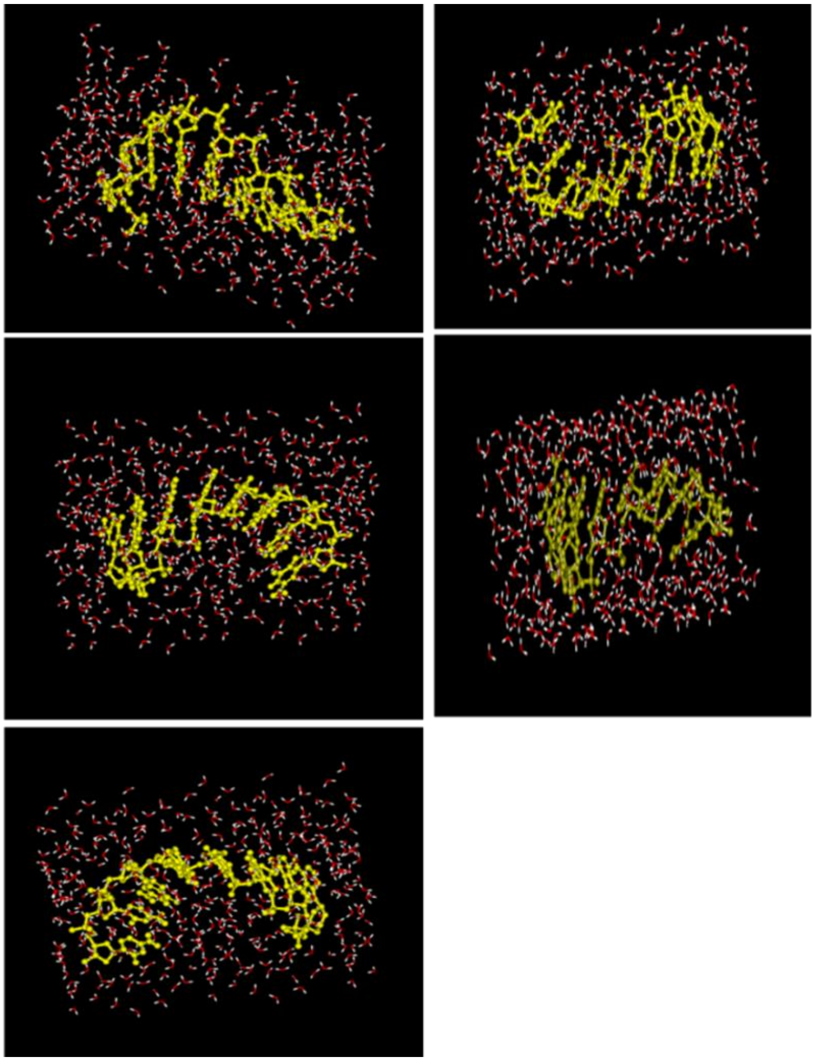

dA_8

dT_8

dG_8

dC_8

$dA_2dT_2dG_2dC_2$

Fig. S4. Molecular modeling of the oligonucleotides dissolved in water. The bright yellow part represents a oligonucleotide molecule， and the square outside yellow is water molecules.
